# Risk factors of pandemic influenza A/H1N1 in a prospective household cohort in the general population: results from the CoPanFlu-France cohort

**DOI:** 10.1111/irv.12294

**Published:** 2014-11-10

**Authors:** Rosemary M Delabre, Nathanael Lapidus, Nicolas Salez, Yohann Mansiaux, Xavier de Lamballerie, Fabrice Carrat

**Affiliations:** a INSERM, UMR_S 1136, Institut Pierre Louis d'Epidémiologie et de Santé PubliqueParis, France; b Sorbonne Universités, UPMC Univ Paris 06, UMR_S 1136, Institut Pierre Louis d'Epidémiologie et de Santé PubliqueParis, France; c Department of Public Health, Hôpital Saint-Antoine, AP-HPParis, France; d UMR_D 190 “Emergence des Pathologies Virales”, IRD French Institute of Research for Development, EHESP French School of Public Health, Aix-Marseille UniversityMarseille, France

**Keywords:** Cohort studies, France, influenza A virus, H1N1 subtype, risk factor

## Abstract

**Background:**

The CoPanFlu-France household cohort was set up in 2009 to identify risk factors of infection by the pandemic A/H1N1 (H1N1pdm09) virus in the general population.

**Objectives:**

To investigate the determinants of infection during the 2010–2011 season, the first complete influenza season of study follow-up for this cohort.

**Patients/Methods:**

Pre- and post-epidemic blood samples were collected for all subjects, and nasal swabs were obtained in all subjects from households where an influenza-like illness was reported. Cases were defined as either a fourfold increase in the serological titer or a laboratory-confirmed H1N1pdm09 on a nasal swab, with either RT-PCR or multiplex PCR. Risk factors for H1N1pdm09 infections were explored, without any pre-specified hypothesis, among 167 individual, collective and environmental covariates via generalized estimating equations modeling. We adopted a multimodel selection procedure to control for model selection uncertainty.

**Results:**

This analysis is based on a sample size of 1121 subjects. The final multivariable model identified one risk factor (history of asthma, OR = 2·17; 95% CI: 1·02–4·62) and three protective factors: pre-epidemic serological titer (OR = 0·51 per doubling of the titer; 95% CI: 0·39–0·67), green tea consumption a minimum of two times a week (OR = 0·39; 95% CI: 0·18–0·84), and proportion of subjects in the household always covering their mouth while coughing/sneezing (OR = 0·93 per 10% increase; 95% CI: 0·86–1·00).

**Conclusion:**

This exploratory study provides further support of previously reported risk factors and highlights the importance of collective protective behaviors in the household. Further analyses will be conducted to explore these findings.

## Introduction

Households are useful epidemiological settings to study influenza infection as an estimated 30% of influenza infections are transmitted within the home.^1^ Risk factors for infection by the novel influenza A/H1N1 pandemic virus (H1N1pdm09) have been studied in households since its identification in spring 2009, and findings have been summarized in a review.^2^ These household studies, however, have largely focused on a limited number of determinants, which mainly rely on sociodemographic characteristics, contact behaviors, or efficacy of prevention measures.^3^–^7^ Furthermore, identified risk factors may not be generalized to the general population as they were largely based on data from case-ascertained studies, in which households including an “index case” are recruited and followed up.

Influenza A/H1N1pdm09 has continued to circulate in conjunction with influenza A/H3N2 and influenza B since the 2009 pandemic. Identification of the determinants of H1N1pdm09 infection, which are not well understood in the context of cocirculation with other seasonal viruses, is important for guiding future public health measures. The CoPanFlu-France cohort, established to study the risk of influenza infection in households in the general population, presents the opportunity to identify the determinants of H1N1pdm09 infection using an large collection of data from both questionnaires and biological samples.^8^

Risk factors associated with high post-epidemic titer following the 2009–2010 pandemic season have previously been reported for this cohort.^9^ Here, we present an analysis relying on the first prospective year of study data to identify factors associated with H1N1pdm09 infections over the 2010–2011 season, exploring a large panel of covariates possibly involved in H1N1pdm09 transmissions.

## Materials and methods

### Study design and participants

Study design and procedures have been previously published.^8^,^9^ From December 2009 to July 2010, a total of 601 families or households (1450 subjects) were included in the CoPanFlu-France cohort. According to the 2009 census, this cohort was overall representative of the French population according to age, sex, household size, urban area size, and socioeconomic distribution.^8^

Over the 2-year follow-up period, annual study visits were conducted by study nurses, which included data collection via questionnaires and blood samples for all enrolled subjects. Additional study visits were programmed when a household declared an influenza-like illness (ILI) or a recent vaccination against influenza via an active automated surveillance system. ILI was defined as fever (≥37·8°C) in addition to cough and/or sore throat. Up to three ILI visits were conducted in the 12 days following symptoms debut; virological samples with nasal swabs were collected for all household members, regardless of their symptom status.

This study focuses on the first year of follow-up (2010–2011) during which the influenza epidemic season in France began on 20 December 2010 and ended 20 February 2011.^10^

We selected participants with a pre-epidemic visit between 9 October 2010 and 6 December 2010 and a post-epidemic visit between 15 June 2011 and 26 January 2012 (1 week before the subsequent 2011–2012 influenza epidemic season^10^). Subjects who had reported a vaccination between the pre- and post-epidemic samples, or within the 2 weeks before the pre-epidemic sample, were excluded from the analysis to avoid misclassification. Households were contacted on a weekly basis from 15 September 2010 to 15 April 2011 via the active automated surveillance system to detect household cases of ILI and trigger ILI visits. Written informed consent was obtained for all enrolled subjects.^8^

### Variables

#### Case definition

Influenza infection with the H1N1pdm09 virus during the 2010–2011 season was the primary outcome for this study. Infected subjects were defined as those who either seroconverted [fourfold increase in hemagglutination inhibition (HI) titer] or had laboratory-confirmed H1N1pdm09 on a nasal swab collected during an ILI visit, using either a monoplex or multiplex molecular detection assay.

#### Baseline covariates

A total of 167 variables concerning characteristics at the individual, household, and environmental levels were collected from questionnaires completed by the subjects at inclusion and organized into five categories: (i) sociodemographic characteristics, habits, and medical history, (ii) preventive measures, (iii) housing characteristics, (iv) social contact information, and (v) geographic characteristics of housing environment. In addition to the covariates studied in the 2009 risk factors analysis,^9^ two new sets of covariates were also considered in the “habits/medical history” category for this analysis: anthropometric information (height, weight, and body mass index) and frequency of tea/coffee consumption. All covariates are described in Tables S1–S5.

As age has been found as a major determinant of influenza infection risk,^11^ we performed sensitivity analyses stratified by age groups (subjects under 15, aged 15–50, and over 50 at inclusion).

Pre-epidemic titer is sometimes studied as a covariate in risk factors analysis.^12^ However, subjects with an elevated pre-epidemic titer may have been previously exposed to the virus (or had a high level of pre-existing cross-immunity). As most risk factors of influenza infection related to a participant's characteristics are likely to be consistent across successive years, pre-epidemic HI titers can be considered in the causal pathway between a risk factor and the outcome, and adjustment on this titer may lead to bias. Additionally, pre-epidemic titer was used to define seroconversions, and it is possible that subjects with a high pre-epidemic titer may be less likely to seroconvert after infection.^13^ We therefore carried out two analyses in parallel, with and without this variable, to assess the impact of this covariate as a risk factor and account for a possible bias.

### Laboratory procedures

The HI titer was determined as the highest dilution providing clear inhibition of hemagglutination in two independent readings.^9^ RT-PCR and multiplex PCR were used to detect viral genome. Viral RNA was extracted from 200 μl of nasal swab eluate using the QIAamp Viral RNA kit (Qiagen, Venlo, Netherlands). TaqMan qRT-PCR was used targeting the hemagglutinin HA gene (SuperScript III Platinum).^9^ The RespiFinder assay was used to simultaneously detect up to 18 respiratory viruses, including the H1N1pdm09.

### Data collection regarding symptoms

During an ILI visit, the presence of the following symptoms was collected in all subjects: cough, runny nose, sore throat, headache, earache, muscle soreness, fatigue, nausea/vomiting, diarrhea, eye redness, and fever (≥37·8°C). This information was checked retrospectively in all subjects, regardless of already reported symptoms, during the following annual visit. In this analysis, we took into account the presence of a symptom if it was reported at least once in a period of 10 days before and after the virus was identified on a swab, or anytime between the blood samples for subjects with serologically defined infection.

### Statistical methods

#### Description of baseline covariates and symptoms

Infected and non-infected subjects were compared using the Mann–Whitney *U*-test for continuous covariates and the Fisher exact test for categorical covariates. Sensitivities of symptoms with their 95% confidence interval (CI) were estimated with the binomial exact test. The pre-epidemic geometric mean titer (GMT) for HI assays was estimated with regression models for interval-censored data with respect for the within-household correlation.^9^ We estimated the impact of post-epidemic blood sample date on seroconversion rate via logistic regression with the use of generalized estimating equations (GEE) with an exchangeable correlation structure accounting for the correlation between responses of subjects living in the same household.

#### Risk factors analysis

Risk factors for infection were estimated with the same method (GEE-based logistic regression). Skewed covariates were log-transformed to obtain normal-like distributions (see Tables S1–S5 for details).

Simultaneous testing of a large amount of covariates yields a type I error rate inflation likely to identify spurious associations. To avoid this drawback, we used a multimodel selection procedure (described in Supporting information) to identify the most relevant covariates among which the final multivariable model should be fitted.^14^ This multimodel selection procedure accounted for model uncertainty, that is, considered that several models could bring insightful information without needing to focus only on the one with the best information criterion.^15^

All covariates were therefore kept in the multivariable models selection, regardless of the univariable analysis, to avoid biases linked to univariable screening.^16^

Multivariable models were selected using the quasi-likelihood under independence model criterion (QIC),^17^ and all multivariable models were assigned a weight according to their relative QIC, which may be interpreted as the weight of evidence in favor of a particular model given the tested subset of models.^18^

A stepwise model selection was then performed, allowing only models with *P* < 0·05 for all factors respectively, among these covariates to obtain the final multivariable model. To account for missing data, this model was averaged from estimations through 30 imputed datasets using Rubin's rule.^19^ This analysis was carried out for the whole sample and stratified by age groups (under 15, between 15 and 50 and over 50 years at inclusion). All analyses were performed using r software version 2.15 (R Foundation for Statistical Computing, Vienna, Austria).

## Results

### Description of infections

Among the 1450 subjects initially included in the cohort, 1318 were followed over the 2010–2011 season. Of the 240 subjects who reported vaccine receipt during the 2010–2011 season, 151 were excluded from the analysis due to reported vaccination within the 2 weeks before, or anytime after, collection of pre-epidemic serological sample (*n* = 108), or inconclusive data regarding a subject's vaccination status or date of vaccination (*n* = 43). No significant differences were found regarding age, sex, medical condition, household size, urban area size, and socioeconomic categories between these subjects and the vaccine recipients included in the cohort. Additionally, 46 other subjects were excluded from the analysis because their blood samples were obtained during the subsequent epidemic period. This analysis is based on a final sample size of 1121 subjects (498 households). A total of 256 ILI visits were carried out in 97 of these households, which included nasal swabs collection in 275 subjects.

Pre-epidemic GMT was 52·8 (95% CI: 50·8–55·0). GMT was higher in subjects under 15 years old at inclusion (GMT: 67·4; 95% CI: 61·2–74·3) than in those aged 15–50 (GMT: 48·2; 95% CI: 45·6–51·1; *P* < 0·0001) or over 50 years old (GMT: 51·7; 95% CI: 48·6–54·9, *P* < 0·0001).

According to our definition, 89 subjects were infected: H1N1pdm09 RNA was detected among 49 subjects and 48 seroconverted (only eight subjects were identified as infected with both methods). The seroconversion rate was 16·3% (95% CI: 7·3–29·7%) in subjects with detected RNA and 3·7% (95% CI: 2·7–5·0%) in others (*P* < 0·001). The number of infections per age group was 24 (10·7%), 36 (7·1%), and 29 (7·4%) for subjects under 15 years, 15–50 years, and over 50 years at inclusion, respectively.

We noted a decrease of the seroconversion rate over time, according to the post-epidemic blood sample date (OR = 0·83 per month; 95% CI: 0·72–0·97).

**Figure 1:**
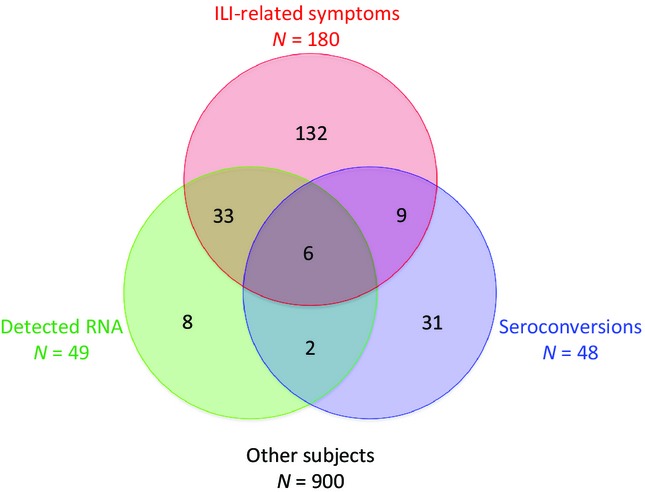
Distribution of reported ILI-related symptoms, seroconversions, and RNA detections in the 1121 studied subjects. ILI, influenza-like illness.

Forty-eight of the 89 infected subjects (53·9%; 95% CI: 43·0–64·6%) reported ILI-related symptoms (Figure 1). This rate was lower in the 48 subjects with seroconversion than in the 41 others: 31·5%; 95% CI: 18·7–46·3% versus 80·5%; 95% CI: 65·1–91·2%, *P* < 0·0001.

Reported symptoms with their respective sensitivities were fever ≥37·8°C (38·2%; 95% CI: 28·1–49·1%), cough (48·3%; 95% CI: 37·6–59·2%), runny nose (43·8%; 95% CI: 33·3–54·7%), sore throat (34·8%; 95% CI: 25·0–45·7%), muscle soreness (32·6%; 95% CI: 23·0–43·3%), fatigue (49·4%; 95% CI: 38·7–60·2%), nausea/vomiting (20·2%; 95% CI: 12·4–30·1%), diarrhea (12·4%; 95% CI: 6·3–21·0%), eye redness (15·7%; 95% CI: 8·9–25·0%), and earache (11·2%; 95% CI: 5·5–19·7%). The sensitivity of CDC-defined ILI was 36·0% (95% CI: 26·1–46·8%).

### Risk factors analysis

Univariable results are given in Tables S1–S5. Analyses with and without pre-epidemic titer as a covariate were performed by selecting 4 and 9 multivariable models (see Tables S6–S18 for details). Among the 13 models, the covariates associated with infections comprised of three potential risk factors (history of asthma, history of cardiovascular disease, and number of children sharing the same bedroom) and nine potential protective factors (pre-epidemic serological titer, 2010–2011 influenza seasonal vaccination, proportion of 2010–2011 influenza vaccine recipients in the household, 2009–2010 pandemic vaccination, reported ILI during the 2009 pandemic wave, number of daily handwashing, mean number of daily handwashing in the household, proportion of household members always covering their mouth while coughing/sneezing, and green tea consumption a minimum of two times a week).

The final multivariable model (Table 1a) fitted from these covariates identified one risk factor (history of asthma, OR = 2·17; 95% CI: 1·02–4·62) and three protective factors: pre-epidemic serological titer (OR = 0·51 per doubling of the titer; 95% CI: 0·39–0·67), green tea consumption a minimum of two times a week (OR = 0·39; 95% CI: 0·18–0·84), and proportion of subjects in the household always covering their mouth while coughing/sneezing (OR = 0·93 per 10% increase; 95% CI: 0·86–1·00). The alpha parameter of this GEE model (intrahousehold correlation of outcomes) was 0·45.

**Table 1:** Multivariable models

	OR	95% CI	*P*
(a) All subjects, *n* = 1121 (89 infected, 1032 non-infected)			
Pre-epidemic titer × 2*	0·51	0·39–0·67	<0·0001
Always covering mouth while coughing/sneezing (household proportion)**	0·93	0·86–1·00	<0·05
History of asthma	2·17	1·02–4·62	<0·05
Green tea consumption***	0·39	0·18–0·84	<0·02
(b) Subjects <15 years at study inclusion, *n* = 505 (36 infected, 469 non-infected)			
Pre-epidemic titer × 2*	0·47	0·28–0·78	<0·01
Always covering mouth while coughing/sneezing (household proportion)**	0·78	0·62–0·97	<0·03
History of asthma	4·18	1·10–15·86	<0·04
(c) Subjects between 15 and 50 years at study inclusion, *n* = 391 (29 infected, 362 non-infected)			
Pre-epidemic titer × 2*	0·47	0·31–0·71	<0·001
History of asthma	3·04	1·08–8·77	<0·04
Green tea consumption***	0·35	0·13–0·98	<0·05
(d) Subjects >50 years at study inclusion, *n* = 225 (24 infected, 201 non-infected)			
Pre-epidemic titer × 2*	0·52	0·32–0·85	<0·01
Always covering mouth while coughing/sneezing (household proportion)	0·87	0·78–0·98	<0·02

** OR per each doubling of the pre-epidemic titer.

****OR per 10% increase in proportion of subjects in the household always covering their mouth while coughing or sneezing.

******Green tea consumption a minimum of twice a week.

Model selection regardless of the pre-epidemic titer identified the same other covariates as associated with the risk of infection: history of asthma (OR = 2·17; 95% CI: 1·21–4·09), green tea consumption a minimum of two times a week (OR = 0·48; 95% CI: 0·25–0·93), and proportion of subjects in the household always covering their mouth while coughing/sneezing (OR = 0·93 per 10% increase; 95% CI: 0·85–1·00).

Final models based on the analysis stratified by age groups did not identify additional factors (Table 1b–d). However, with the exception of pre-epidemic titer, which was associated with infections in all age groups, the final models retained different covariates: asthma was identified as a possible risk factor in subjects under 15 and 15–50 years only, proportion of household members always covering their mouth while coughing/sneezing was identified only for subjects under 15 and those over 50 years, whereas green tea consumption was associated with infections for subjects aged 15–50 years only.

## Discussion

### Factors associated with H1N1pdm09 infection

The CoPanFlu cohort was designed to collect a large amount of covariates studied simultaneously in order to identify those likely to be involved in the transmission of influenza. We previously identified factors associated with high post-pandemic serological titer in a retrospective, nested case–control analysis conducted in this cohort:^9^ young age, chronic obstructive pulmonary disease, asthma, social contacts at school, and the use of public transportation were identified as possible risk factors, whereas presence of an air humidifier in the living room was a possible protective factor. In the present analysis, we identified factors associated with the H1N1pdm09 infection during the 2010–2011 influenza season. Our results support previously reported risk factors for this season, in addition to identifying a potentially protective role of collective behaviors among household members.

High pre-seasonal titers observed in this study were protective against H1N1pdm09 infection, as previously reported in other studies.^12^,^20^ This result is not surprising as the HI titer is known as a major correlate of protection.^21^

The household environment has been identified as an important factor in the transmission of influenza.^22^ The high correlation between outcomes of subjects living in the same household (alpha parameter of the GEE model) was expected as these individuals share some risk factors in addition to environmental (within-household) exposure to influenza viruses.

Household efficacy studies of non-pharmaceutical interventions such as regular handwashing/hygiene and use of face masks suggest a protective benefit if implemented in a timely manner after symptoms are observed in the index patient.^6^ Public health communications, such as the CDC's “Cover your Cough” campaign, heavily stress the importance of respiratory hygiene and cough etiquette to stop influenza transmission.^23^ We studied several preventive behaviors at the household level, taking into account the impact of other members’ behaviors for households of at least two subjects. However, only the proportion of household members covering their mouth while coughing was found to be a protective factor against H1N1pdm09 infection.

Asthma was already identified as a possible risk factor for H1N1pdm09 infection in this cohort, regarding the 2009 pandemic.^9^ Severity of infections observed in asthmatics^24^ may be due to a greater immune response,^25^ increasing the probability of virus detection (ILI visits). Alternatively, asthmatic subjects may have an increased susceptibility for H1N1pdm09 infection;^26^ regardless of the virological definition of infection, the seroconversion rate is higher in asthmatic subjects (11·7% versus 3·7%, *P* < 0·01).

Effects of green tea catechins and theanine to prevent ILI have been demonstrated in a double-blind randomized controlled trial,^27^ and in another study, daily green tea consumption has been associated with a lower incidence of influenza infection.^28^ In our study, the absence of an association between infection and either coffee or black tea consumption as negative control exposures strengthens the relevance of the association with green tea. As for the other identified risk factors in our exploratory analysis, the effects of green tea on influenza infection risk in this population would need to be confirmed in a dedicated study, such as a randomized controlled trial with a more detailed collection of frequency and quantity of consumption.

Several other factors were identified as relevant by the multimodel selection; however, they did not meet the significance threshold in the final multivariable models. Our study was an exploratory analysis of risk factors for infection, and such factors should be considered as possibly insightful results given a hypothesis-generating objective. For example, we note the presence of several covariates regarding household characteristics: proportion of 2010–2011 vaccine recipients in the household and mean number of daily handwashing in the household. These covariates, in addition to proportion of household members always covering their mouth while coughing, highlight the potential importance of collective behaviors in the household to limit transmissions at an individual level.

Infection rate was slightly higher in subjects under 15 years old, but the difference with other age groups was not significant (*P* = 0·10). Contrary to the first pandemic wave in 2009,^9^ age was not identified as a factor associated with infections in the multimodel selection. A large proportion of young subjects may have been infected during the 2009 wave, as evidenced by their high pre-epidemic GMT. Furthermore, reported ILI during the 2009 pandemic wave was associated with a lower infection rate in the multimodel selection (this association disappeared after adjustment on the pre-epidemic titer).

Other evidence pointing to a change in the susceptible population during the second season is the absence of a factor related to contact behaviors. Contacts at school, which is very specific to the susceptible population during the first season,^9^ were not associated with infection in this present analysis likely because the susceptible population during the second year did not have such specific contact behaviors. However, it is possible that either the definition of contact type^9^ or the duration was not a relevant measure of contacts likely to favor virus transmission. Regardless, this negative result is a reminder that measures to limit direct contacts may have minimal impact on transmission via aerosols^29^ or fomites.^30^

## Strengths and limitations

To our knowledge, this cohort is the first attempt to simultaneously study such a large amount of variables regarding individual, household, and environmental determinants of influenza in the general population. The major challenge in this exploratory analysis was to identify the most relevant factors while avoiding spurious associations. The multimodel selection allowed us to select a limited set of covariates according to their relevancy in multivariable analysis, thus avoiding the biases linked to univariable screening. The final model selection was therefore conducted in a restricted number of covariates, with a type I error comparable to most epidemiological studies. Multimodel selection also accounted for model uncertainty, which may be a more appropriate method of model selection compared to those traditionally used in exploratory analyses.^14^

One limitation in this study is linked to the case definition. Although the fourfold increase definition of seroconversions is widely used, its lack of sensitivity has been demonstrated.^31^ Moreover, post-infectious HI titers are known to decrease over time.^9^,^32^ The negative association between the post-epidemic blood sample date and seroconversion rate may partly explain the low seroconversion rate (19·5%) in subjects with detected RNA; more infections may have been identified by seroconversion if blood samples had been collected right after the epidemic. This may have impaired the power of our risk factor analysis and therefore limited the likeliness to identify factors associated with infections. However, as we found no association between the delay between blood samples and main sociodemographic factors or medical characteristics, we think this timing issue unlikely to have involved confounding.

Specificity of seroconversions might also be questionable but as no other A/H1N1 virus was identified during this season, cross-reactions are unlikely in this analysis. Virological samples were not obtained in households experiencing only asymptomatic infections (concerning up to 67% of seasonal influenza infections^33^), which may explain the differences in symptomatic rates according to the diagnostic method. Interestingly, the best estimate of the true symptomatic rate may be the one relying on subjects with seroconversion (31·5%; CI: 18·7%, 46·3%), a result that is comparable to a recently published estimation regarding different seasonal strains (23%; 95% CI: 13–34).^34^

Finally, influenza A/H1N1pdm09 circulated in the presence of other viral strains during the 2010–2011 influenza season: influenza A/H3N2 (8·1%) and influenza B (48%).^35^ In our study, RT-PCR and multiplex PCR were performed on all samples and identified 37 influenza B infections (including 10 coinfections with H1N1pdm09) and 44 seasonal influenza A infections (including 30 coinfections with H1N1pdm09) of the 1121 studied subjects. At least 38 subjects infected with seasonal influenza did not meet the case definition and were considered as “non-infected”; therefore, this analysis identified risk factors associated with H1N1pdm09 infections only. As a result of these limitations in laboratory procedures, we can assume that our case definition may have failed to identify some infected subjects, whereas its specificity raises no major issue.

This exploratory risk factor analysis used a large amount of covariates without any pre-specified hypothesis. Although this data-driven approach provides an easy interpretation of results, it relies on the assumption that risk factors are directly associated with infections. Using the same database, we have also performed a parallel study using a hypothesis-driven approach in which data are used to support postulated causal relationships in a structural model to identify factors associated with infections.^36^ While both approaches have their respective strengths and limitations, they have allowed us to exploit the richness of the CoPanFlu-France cohort data; these complementary analyses help further understanding of the determinants of influenza infection.

## Conclusion

The data and biological samples collected within the CoPanFlu-France cohort study have allowed a comprehensive analysis of the determinants of H1N1pdm09 infection during the 2010–2011 season within households in the general population. The broad scope of this exploratory analysis has permitted to identify factors expected from previously reported studies (pre-epidemic serologic titer, asthma, and green tea) and to highlight the potential importance of collective preventive behaviors in the household, including the proportion of household covering their mouth when coughing which was associated with H1N1pdm09 in the final multivariable model. Additional analyses are being conducted for this household cohort whose follow-up ended in late 2012 to provide complimentary insights for investigating risk factors of infectious disease.

## Supporting Information

**Data S1.** Description and univariable analyses for all covariates (Tables S1–S5).

**Data S2.** Multivariable selection procedure and results (Tables S6–S18).
