# Supplementary material for: Risk factors of pandemic influenza A/H1N1 in a prospective household cohort in the general population: results from the CoPanFlu-France cohort
Source: Influenza Other Respir Viruses. 2014 Nov 10;9(1):43–50. doi: 10.1111/irv.12294 (PMC4280818; doi:10.1111/irv.12294)
Supplement: Supplementary file 1 — Data S1. Description and univariable analyses for all covariates (Tables S1–S5). [file irv0009-0043-sd1.docx]

**Risk factors of pandemic influenza A/H1N1 in a prospective household cohort in the general population: results from the CoPanFlu-France cohort**

Supplementary tables S1–S5: description and univariable analyses for all covariates

- Table S1: Socio-demographic characteristics, habits and medical history
- Table S2: Preventive measures
- Table S3: Environmental characteristics of the housing
- Table S4: Nature and daily duration of meetings
- Table S5: Geographic area

Distributions of covariates are given as number (proportion) or median [interquartile range (IQR)] for binary or quantitative variables respectively. Quantitative covariates noted with an asterisk (*) were log-transformed for the regression analysis (log2 for the pre-epidemic titer, natural log for other covariates). Information regarding hot beverage consumption and preventive behaviors were collected as ordinal covariates: several (at least 2) times a week / several (at least 2) times a month / rarely / never and always / often / rarely / never, respectively. These variables were dichotomized to build binary covariates by grouping adjacent categories: everyday / several times a week vs. lower frequency and always vs. lower frequency, respectively. Thresholds were chosen to ensure a number of subjects in each category likely to provide enough power in the risk factor analysis. Covariates regarding “all subjects in the household” are defined as the proportion or mean value in the household for binary or quantitative variables respectively. Distributions in infected and non-infected subjects are compared with a Fisher or Mann-Whitney test for binary or quantitative covariates respectively. The p-value of the test is reported in the P (diff) column.

Odds ratios (OR) for the association with the infected status (GEE univariable logistic regression) are estimated with their 95% confidence intervals (CI) from observed values in the original dataset. OR for proportions are given for a 0% to 100% increase. The associated p-value is reported in the P (OR) column.

**Table S1. Socio-demographic characteristics, habits and medical history**

| **Socio-demographic characteristic** |  |  |  |  |  |  |  |  |
| --- | --- | --- | --- | --- | --- | --- | --- | --- |
|  | ***Missing values (N)*** | ***Distribution (all)*** | ***Distribution in infected subjects*** | ***Distribution in non-infected subjects*** | ***P (diff)*** | ***OR*** | ***(95% CI)*** | ***P (OR)*** |
| Sex = male | 0 | 520 (46.4%) | 47 (52.8%) | 473 (45.8%) | 0.22 | 1.32 | 0.88, 1.99 | 0.18 |
| Age at inclusion (distributions: years, OR: per 10 years) | 0 | 40.5 [18.4, 56.4] | 35.8 [11.3, 54.1] | 41.1 [18.9, 56.6] | < 0.05 | 0.90 | 0.80, 1.01 | 0.07 |
| - 0 to 15 years old |  | 225 (20.0%) | 24 (27.0%) | 201 (19.5%) |  | 0.97 | 0.91, 1.03 | 0.25 |
| - 15 to 50 years old |  | 505 (45.0%) | 36 (40.5%) | 469 (45.4%) | 0.24 | 1.00 | (ref) |  |
| - Over 50 years old |  | 391 (34.9%) | 29 (32.6%) | 362 (35.1%) |  | 0.97 | 0.92, 1.01 | 0.15 |
| Has a professional activity | 0 | 543 (48.4%) | 45 (50.6%) | 498 (48.3%) | 0.74 | 1.10 | 0.70, 1.73 | 0.69 |
| Professional activity: |  |  |  |  |  |  |  |  |
| - Primarily in confined space | 0 | 396 (35.3%) | 30 (33.7%) | 366 (35.5%) | 0.82 | 0.93 | 0.58, 1.47 | 0.74 |
| - Primarily outdoors | 0 | 98 (8.7%) | 12 (13.5%) | 86 (8.3%) | 0.11 | 1.71 | 0.90, 3.26 | 0.10 |
| - Involves contact with children | 0 | 169 (15.1%) | 11 (12.4%) | 158 (15.3%) | 0.54 | 0.78 | 0.41, 1.47 | 0.44 |
| - Involves contact with ill people | 0 | 116 (10.3%) | 13 (14.6%) | 103 (10.0%) | 0.20 | 1.54 | 0.82, 2.91 | 0.18 |
|  |  |  |  |  |  |  |  |  |

| **Metrics** |  |  |  |  |  |  |  |  |
| --- | --- | --- | --- | --- | --- | --- | --- | --- |
| Height | 51 | 167 [158, 174] | 166 [148, 172] | 167 [158, 174] | 0.06 | 0.99 | 0.98, 0.99 | < 0.003 |
| Weight | 49 | 66.0 [53.0, 80.0] | 68.5 [45.0, 79.5] | 66.0 [54.0, 80.0] | 0.47 | 0.99 | 0.98, 1.00 | 0.18 |
| Body mass index | 0 | 23.0 [19.4, 26.5] | 23.3 [18.0, 27.8] | 23.0 [19.5, 26.4] | 0.71 | 1.01 | 0.98, 1.04 | 0.68 |

| **Medical history** |  |  |  |  |  |  |  |  |
| --- | --- | --- | --- | --- | --- | --- | --- | --- |
| Self-rated good health in the last 12 months | 16 | 698 (63.2%) | 54 (60.7%) | 644 (63.4%) | 0.65 | 0.89 | 0.56, 1.42 | 0.63 |
| Past or ongoing chronic condition | 0 | 424 (37.8%) | 36 (40.4%) | 388 (37.6%) | 0.65 | 1.13 | 0.69, 1.85 | 0.64 |
| Diabetes | 0 | 46 (4.1%) | 5 (5.6%) | 41 (4.0%) | 0.40 | 1.44 | 0.54, 3.83 | 0.47 |
| History of asthma | 0 | 77 (6.9%) | 12 (13.5%) | 65 (6.3%) | < 0.02 | 2.32 | 1.17, 4.6 | < 0.02 |
| Ongoing asthma | 0 | 44 (3.9%) | 6 (6.7%) | 38 (3.7%) | 0.15 | 1.89 | 0.7, 5.13 | 0.21 |
| Chronic obstructive pulmonary disease | 0 | 34 (3.0%) | 4 (4.5%) | 30 (2.9%) | 0.34 | 1.57 | 0.55, 4.51 | 0.40 |
| Hypertension | 0 | 121 (10.8%) | 10 (11.2%) | 111 (10.8%) | 0.86 | 1.05 | 0.51, 2.17 | 0.89 |
| Chronic renal disease | 0 | 9 (0.8%) | 1 (1.1%) | 8 (0.8%) | 0.53 | 1.45 | 0.18, 12.01 | 0.73 |
| Systemic disease | 0 | 28 (2.5%) | 2 (2.2%) | 26 (2.5%) | 1.00 | 0.89 | 0.21, 3.81 | 0.87 |
| Chronic respiratory insufficiency | 0 | 10 (0.9%) | 2 (2.2%) | 8 (0.8%) | 0.19 | 2.94 | 0.63, 13.74 | 0.17 |
| Cardiac insufficiency | 0 | 18 (1.6%) | 1 (1.1%) | 17 (1.6%) | 1.00 | 0.68 | 0.09, 5.07 | 0.71 |
| Cardiac arrhythmia | 0 | 47 (4.2%) | 8 (9.0%) | 39 (3.8%) | < 0.03 | 2.51 | 1.05, 6.02 | < 0.04 |
| Valvular heart disease | 0 | 13 (1.2%) | 1 (1.1%) | 12 (1.2%) | 1.00 | 0.97 | 0.13, 7.41 | 0.97 |
| Coronary insufficiency | 0 | 28 (2.5%) | 4 (4.5%) | 24 (2.3%) | 0.27 | 1.98 | 0.68, 5.71 | 0.21 |
| History of myocardial infarction | 0 | 11 (1.0%) | 1 (1.1%) | 10 (1.0%) | 0.60 | 1.16 | 0.15, 8.82 | 0.89 |
| History of stroke | 0 | 11 (1.0%) | 1 (1.1%) | 10 (1.0%) | 0.60 | 1.16 | 0.15, 9.23 | 0.89 |
| Cancer or leukemia | 0 | 36 (3.2%) | 3 (3.4%) | 33 (3.2%) | 0.76 | 1.06 | 0.32, 3.48 | 0.93 |
| Chemotherapy | 0 | 11 (1.0%) | 2 (2.2%) | 9 (0.9%) | 0.22 | 2.61 | 0.57, 11.99 | 0.22 |
| Radiotherapy | 0 | 14 (1.2%) | 2 (2.2%) | 12 (1.2%) | 0.31 | 1.95 | 0.43, 8.84 | 0.38 |
| Chronic neuromuscular disorder | 0 | 23 (2.1%) | 3 (3.4%) | 20 (1.9%) | 0.42 | 1.77 | 0.52, 5.99 | 0.36 |
| Multiple sclerosis | 0 | 7 (0.6%) | 2 (2.2%) | 5 (0.5%) | 0.10 | 4.72 | 0.91, 24.4 | 0.06 |
| Allergy (any) | 0 | 12 (1.1%) | 1 (1.1%) | 11 (1.1%) | 1.00 | 1.05 | 0.13, 8.35 | 0.96 |
| Any respiratory disease | 0 | 105 (9.4%) | 14 (15.7%) | 91 (8.8%) | < 0.04 | 1.93 | 1.02, 3.64 | < 0.05 |
| Migraine, headaches | 0 | 3 (0.3%) | 1 (1.1%) | 2 (0.2%) | 0.22 | 5.85 | 0.53, 64.3 | 0.15 |
| Breast cancer | 0 | 7 (0.6%) | 1 (1.1%) | 6 (0.6%) | 0.44 | 1.94 | 0.24, 16.04 | 0.54 |
| Prostate cancer | 0 | 6 (0.5%) | 1 (1.1%) | 5 (0.5%) | 0.39 | 2.33 | 0.27, 20.28 | 0.44 |
| Immunodepression | 0 | 76 (6.8%) | 7 (7.9%) | 69 (6.7%) | 0.66 | 1.19 | 0.52, 2.72 | 0.68 |
| Any cardiovascular disease | 0 | 161 (14.4%) | 17 (19.1%) | 144 (14%) | 0.21 | 1.46 | 0.78, 2.72 | 0.24 |
|  |  |  |  |  |  |  |  |  |

| **History of ILI** |  |  |  |  |  |  |  |  |
| --- | --- | --- | --- | --- | --- | --- | --- | --- |
| - Season 2009-2010 | 40 | 87 (8.0%) | 7 (8.0%) | 80 (8.0%) | 1.00 | 1.00 | 0.42, 2.41 | 1.00 |
| - Season 2008-2009 | 0 | 184 (16.4%) | 15 (16.9%) | 169 (16.4%) | 0.88 | 1.04 | 0.56, 1.92 | 0.91 |
| - Season 2007-2008 | 0 | 111 (9.9%) | 10 (11.2%) | 101 (9.8%) | 0.71 | 1.17 | 0.57, 2.37 | 0.67 |
| - Season 2006-2007 | 0 | 101 (9.0%) | 8 (9.0%) | 93 (9.0%) | 1.00 | 1.00 | 0.46, 2.17 | 0.99 |
|  |  |  |  |  |  |  |  |  |
| **Pre-epidemic titer*** (OR per doubling) | 0 | 1/40 [1/20, 1/40] | 1/20 [1/20, 1/40] | 1/40 [1/20, 1/40] | < 0.001 | 0.52 | 0.39, 0.68 | < 0.001 |
|  |  |  |  |  |  |  |  |  |
| **Smoking habits** |  |  |  |  |  |  |  |  |
| History of smoking | 0 | 434 (38.7%) | 32 (36.0%) | 402 (39.0%) | 0.65 | 0.88 | 0.53, 1.45 | 0.62 |
| Current smoker | 0 | 223 (19.9%) | 17 (19.1%) | 206 (20.0%) | 1.00 | 0.95 | 0.49, 1.82 | 0.87 |
| Smoke inside home | 0 | 124 (11.1%) | 10 (11.2%) | 114 (11.0%) | 1.00 | 1.02 | 0.42, 2.5 | 0.97 |
|  |  |  |  |  |  |  |  |  |
| **Coffee/tea consumption (several times a week)** |  |  |  |  |  |  |  |  |
| Coffee | 1 | 691 (61.7%) | 49 (55.1%) | 642 (62.3%) | 0.21 | 0.74 | 0.49, 1.13 | 0.17 |
| Black tea | 1 | 142 (12.7%) | 12 (13.5%) | 130 (12.6%) | 0.74 | 1.08 | 0.55, 2.14 | 0.82 |
| Green tea | 1 | 193 (17.2%) | 8 (9.0%) | 185 (17.9%) | < 0.03 | 0.45 | 0.22, 0.93 | < 0.04 |
|  |  |  |  |  |  |  |  |  |
| **Smoking habits in all subjects of the household** |  |  |  |  |  |  |  |  |
| History of smoking | 0 | 0.33 [0.0, 0.5] | 0.33 [0.0, 0.67] | 0.33 [0.0, 0.5] | 0.91 | 1.10 | 0.45, 2.69 | 0.83 |
| Current smoker | 0 | 0.0 [0.0, 0.33] | 0.0 [0.0, 0.33] | 0.0 [0.0, 0.33] | 0.68 | 1.09 | 0.39, 3.08 | 0.87 |
| Smokes inside home | 0 | 0.0 [0.0, 0.0] | 0.0 [0.0, 0.0] | 0.0 [0.0, 0.0] | 0.79 | 1.42 | 0.45, 4.48 | 0.55 |

**Table S2. Preventive measures**

|  | ***Missing values (N)*** | ***Distribution (all)*** | | ***Distribution in infected subjects*** | ***Distribution in non-infected subjects*** | ***P (diff)*** | ***OR*** | ***(95% CI)*** | | ***P (OR)*** | |
| --- | --- | --- | --- | --- | --- | --- | --- | --- | --- | --- | --- |
| **Vaccination** |  |  |  | |  |  |  |  |  | |  |
| Influenza: |  |  |  | |  |  |  |  |  | |  |
| - Current season (2010) before epidemic | 0 | 102 (9.1%) | 6 (6.7%) | | 96 (9.3%) | 0.56 | 0.70 | 1.40, 0.36 | 0.49 | |  |
| - Pandemic vaccination (2009) | 2 | 126 (11.3%) | 5 (5.6%) | | 121 (11.7%) | 0.08 | 0.45 | 0.18, 1.09 | 0.08 | |  |
| - Seasonal vaccination 2009-2010 | 2 | 163 (14.6%) | 10 (11.2%) | | 153 (14.9%) | 0.43 | 0.73 | 0.34, 1.53 | 0.40 | |  |
| - Any season from 2006-2007 to 2008-2009 | 8 | 193 (17.3%) | 14 (15.7%) | | 179 (17.5%) | 0.77 | 0.88 | 0.46, 1.68 | 0.70 | |  |
| Anti-pneumococcal vaccination | 36 | 60 (5.5%) | 3 (3.6%) | | 57 (5.7%) | 0.62 | 0.61 | 0.19, 1.94 | 0.41 | |  |
|  |  |  |  | |  |  |  |  |  | |  |
| **Hygiene measures** |  |  |  | |  |  |  |  |  | |  |
| Always covers mouth while coughing or sneezing | 14 | 547 (49.4%) | 33 (37.5%) | | 514 (50.4%) | < 0.03 | 0.59 | 0.36, 0.97 | < 0.04 | |  |
| Daily frequency of handwashing  (with soap or hand sanitizer) | 30 | 5 [3, 10] | 5 [3, 10] | | 5 [3, 10] | 0.37 | 0.98 | 0.94, 1.03 | 0.50 | |  |
| Always washes hands after coughing or sneezing | 15 | 91 (8.2%) | 11 (12.5%) | | 80 (7.9%) | 0.15 | 1.67 | 0.88, 3.17 | 0.11 | |  |
|  |  |  |  | |  |  |  |  |  | |  |
| **Preventive measures in all subjects of the household** |  |  |  | |  |  |  |  |  | |  |
| Influenza vaccination |  |  |  | |  |  |  |  |  | |  |
| - Current season (2010) before epidemic | 0 | 0.0 [0.0, 0.0] | 0.0 [0.0, 0.0] | | 0.0 [0.0, 0.0] | 0.40 | 0.73 | 1.34, 0.40 | 0.59 | |  |
| - Pandemic vaccination (2009) | 0 | 0.0 [0.0, 0.0] | 0.0 [0.0, 0.0] | | 0.0 [0.0, 0.0] | < 0.03 | 0.36 | 0.12, 1.08 | 0.07 | |  |
| - Seasonal vaccination 2009-2010 | 0 | 0.0 [0.0, 0.2] | 0.0 [0.0, 0.0] | | 0.0 [0.0, 0.2] | 0.16 | 0.70 | 0.28, 1.70 | 0.43 | |  |
| - Any season from 2006-2007 to 2008-2009 | 0 | 0.0 [0.0, 0.25] | 0.0 [0.0, 0.25] | | 0.0 [0.0, 0.26] | 0.81 | 0.95 | 0.43, 2.06 | 0.89 | |  |
| Always covers mouth while coughing or sneezing | 0 | 0.5 [0.0, 1.0] | 0.2 [0.0, 0.75] | | 0.5 [0.17, 1.0] | < 0.005 | 0.44 | 0.21, 0.93 | < 0.04 | |  |
| Daily frequency of handwashing  (with soap or hand sanitizer) | 0 | 5.9 [4.0, 8.3] | 5.5 [4.3, 7.0] | | 6.0 [4.0, 8.6] | 0.25 | 0.96 | 0.90, 1.02 | 0.21 | |  |
| Always washes hands after coughing or sneezing | 0 | 0.0 [0.0, 0.0] | 0.0 [0.0, 0.17] | | 0.0 [0.0, 0.0] | 0.05 | 1.82 | 0.63, 5.27 | 0.27 | |  |

**Table S3. Environmental characteristics of the housing**

|  | ***Missing values (N)*** | ***Distribution (all)*** | | ***Distribution in infected subjects*** | | ***Distribution in non-infected subjects*** | | ***P (diff)*** | ***OR*** | ***(95% CI)*** | | ***P (OR)*** | |
| --- | --- | --- | --- | --- | --- | --- | --- | --- | --- | --- | --- | --- | --- |
| **General characteristics** |  | |  | |  | |  |  |  |  |  | |  |
| Number of subjects in the household | 0 | | 3 [2, 4] | | 3 [2, 4] | | 3 [2, 4] | 0.85 | 1.03 | 0.84, 1.26 | 0.81 | |  |
| Number of children (<15 years) in the household | 0 | | 0 [0, 2] | | 0 [0, 2] | | 0 [0, 2] | 0.54 | 1.04 | 0.76, 1.44 | 0.79 | |  |
| Number of other children in the household | 0 | | 0 [0, 1] | | 0 [0, 1] | | 0 [0, 1] | 0.26 | 0.98 | 0.65, 1.49 | 0.94 | |  |
| Habitation = house | 0 | | 735 (65.6%) | | 51 (57.3%) | | 684 (66.3%) | 0.10 | 0.68 | 0.4, 1.17 | 0.17 | |  |
| Surface area of habitation (m²)* | 15 | | 100 [80, 135] | | 99 [79.5, 124] | | 100 [80, 134] | 0.56 | 0.91 | 0.52, 1.6 | 0.75 | |  |
| Surface area of habitation per subject (m²)* | 15 | | 36.7 [25, 21.6] | | 35 [25, 50] | | 37.5 [25.5, 50] | 0.79 | 0.87 | 0.52, 1.44 | 0.59 | |  |
| Number of rooms in habitation | 21 | | 5 [4, 6] | | 5 [4, 5.75] | | 5 [4, 6] | < 0.03 | 0.87 | 0.76, 0.99 | < 0.04 | |  |
| Number of rooms per subject in habitation | 21 | | 1.67 [1.25, 2.50] | | 1.5 [1.06, 2.50] | | 1.67 [1.25, 2.50] | 0.12 | 0.82 | 0.63, 1.07 | 0.15 | |  |
| Mechanical ventilation | 26 | | 596 (54.4%) | | 42 (48.8%) | | 554 (54.9%) | 0.31 | 0.78 | 0.46, 1.33 | 0.37 | |  |
| Presence of thermostat | 0 | | 584 (52.1%) | | 45 (50.6%) | | 539 (52.2%) | 0.83 | 0.94 | 0.55, 1.59 | 0.80 | |  |
| Number of bathrooms | 0 | | 1 [1, 2] | | 1 [1, 1] | | 1 [1, 2] | 0.48 | 0.91 | 0.58, 1.43 | 0.69 | |  |
| Number of toilets | 0 | | 2 [2, 2] | | 2 [2, 2] | | 2 [2, 2] | 0.21 | 0.79 | 0.52, 1.2 | 0.27 | |  |
| Presence of garden/backyard or land surrounding habitation | 0 | | 886 (79%) | | 66 (74.2%) | | 820 (79.5%) | 0.28 | 0.74 | 0.39, 1.41 | 0.36 | |  |
|  |  | |  | |  | |  |  |  |  |  | |  |
| **Presence of animals inside habitation** |  | |  | |  | |  |  |  |  |  | |  |
| Any | 0 | | 588 (52.5%) | | 50 (56.2%) | | 538 (52.1%) | 0.51 | 1.18 | 0.69, 2 | 0.55 | |  |
| Dogs | 0 | | 296 (26.4%) | | 25 (28.1%) | | 271 (26.3%) | 0.71 | 1.10 | 0.59, 2.03 | 0.77 | |  |
| Cats | 0 | | 332 (29.6%) | | 32 (36%) | | 300 (29.1%) | 0.18 | 1.37 | 0.78, 2.39 | 0.27 | |  |
| Domestic rodents | 0 | | 93 (8.3%) | | 7 (7.9%) | | 86 (8.3%) | 1.00 | 0.94 | 0.31, 2.83 | 0.91 | |  |
| Birds | 0 | | 49 (4.4%) | | 4 (4.5%) | | 45 (4.4%) | 1.00 | 1.03 | 0.22, 4.91 | 0.97 | |  |
|  |  | |  | |  | |  |  |  |  |  | |  |
|  |  | |  | |  | |  |  |  |  |  | |  |

| **Kitchen** |  |  |  |  |  |  |  |  |  |
| --- | --- | --- | --- | --- | --- | --- | --- | --- | --- |
| Presence of a kitchen range hood in the kitchen | 0 | 837 (74.7%) | 68 (76.4%) | 769 (74.5%) | 0.80 | 1.11 | 0.59, 2.09 | 0.75 |  |
| Filtration of area | 0 | 598 (53.3%) | 36 (40.4%) | 562 (54.5%) | < 0.02 | 0.57 | 0.34, 0.96 | < 0.04 |  |
| Presence of dishwasher | 0 | 782 (69.8%) | 69 (77.5%) | 713 (69.1%) | 0.12 | 1.54 | 0.83, 2.88 | 0.17 |  |
| Heating: |  |  |  |  |  |  |  |  |  |
| - Floor heating | 0 | 146 (13%) | 12 (13.5%) | 134 (13.0%) | 0.87 | 1.04 | 0.41, 2.66 | 0.93 |  |
| - Wall heating unit | 0 | 868 (77.4%) | 63 (70.8%) | 805 (78.0%) | 0.14 | 0.68 | 0.37, 1.27 | 0.23 |  |
| - Central heating | 0 | 546 (48.7%) | 46 (51.7%) | 500 (48.4%) | 0.58 | 1.14 | 0.67, 1.92 | 0.63 |  |
| - Electric heating | 0 | 332 (29.6%) | 22 (24.7%) | 310 (30%) | 0.33 | 0.76 | 0.44, 1.33 | 0.34 |  |
| - Fuel heating | 0 | 173 (15.4%) | 10 (11.2%) | 163 (15.8%) | 0.29 | 0.67 | 0.29, 1.55 | 0.36 |  |
| - Fireplace/chimney heating | 0 | 122 (10.9%) | 7 (7.9%) | 115 (11.1%) | 0.48 | 0.68 | 0.32, 1.47 | 0.33 |  |
| - Wood heating stove | 0 | 66 (5.9%) | 8 (9.0%) | 58 (5.6%) | 0.23 | 1.66 | 0.63, 4.4 | 0.31 |  |
| - Gas heating | 0 | 474 (42.3%) | 42 (47.2%) | 432 (41.9%) | 0.37 | 1.24 | 0.73, 2.11 | 0.42 |  |
| Flooring: |  |  |  |  |  |  |  |  |  |
| - Tiles flooring | 0 | 927 (82.7%) | 75 (84.3%) | 852 (82.6%) | 0.77 | 1.13 | 0.48, 2.65 | 0.78 |  |
| - Linoleum flooring | 0 | 165 (14.7%) | 15 (16.9%) | 150 (14.5%) | 0.53 | 1.19 | 0.54, 2.65 | 0.67 |  |
| - Carpeting or rug/mat flooring | 0 | 38 (3.4%) | 2 (2.2%) | 36 (3.5%) | 0.76 | 0.64 | 0.15, 2.64 | 0.53 |  |
| - Hardwood flooring | 0 | 192 (17.1%) | 14 (15.7%) | 178 (17.2%) | 0.88 | 0.9 | 0.44, 1.81 | 0.76 |  |
|  |  |  |  |  |  |  |  |  |  |
| **Living room** |  |  |  |  |  |  |  |  |  |
| Presence of air humidifier | 0 | 50 (4.5%) | 5 (5.6%) | 45 (4.4%) | 0.59 | 1.31 | 0.45, 3.75 | 0.62 |  |
|  |  |  |  |  |  |  |  |  |  |
| Heating: |  |  |  |  |  |  |  |  |  |
| - Floor heating | 0 | 145 (12.9%) | 16 (18%) | 129 (12.5%) | 0.14 | 1.53 | 0.68, 3.47 | 0.3 |  |
| - Wall heating unit | 0 | 832 (74.2%) | 62 (69.7%) | 770 (74.6%) | 0.31 | 0.78 | 0.43, 1.43 | 0.42 |  |
| - Central heating | 0 | 516 (46%) | 40 (44.9%) | 476 (46.1%) | 0.91 | 0.95 | 0.56, 1.62 | 0.86 |  |
| - Electric heating | 0 | 307 (27.4%) | 20 (22.5%) | 287 (27.8%) | 0.32 | 0.75 | 0.42, 1.33 | 0.33 |  |
| - Fuel heating | 0 | 171 (15.3%) | 12 (13.5%) | 159 (15.4%) | 0.76 | 0.86 | 0.36, 2.02 | 0.72 |  |
| - Fireplace/chimney heating | 0 | 169 (15.1%) | 15 (16.9%) | 154 (14.9%) | 0.64 | 1.16 | 0.63, 2.12 | 0.64 |  |
| - Wood heating stove | 0 | 75 (6.7%) | 7 (7.9%) | 68 (6.6%) | 0.66 | 1.21 | 0.41, 3.62 | 0.73 |  |
| - Gas heating | 0 | 465 (41.5%) | 41 (46.1%) | 424 (41.1%) | 0.37 | 1.22 | 0.72, 2.08 | 0.45 |  |
|  |  |  |  |  |  |  |  |  |  |

| Flooring: |  |  |  |  |  |  |  |  |  |
| --- | --- | --- | --- | --- | --- | --- | --- | --- | --- |
| - Tiles flooring | 0 | 689 (61.5%) | 56 (62.9%) | 633 (61.3%) | 0.82 | 1.07 | 0.61, 1.88 | 0.82 |  |
| - Linoleum flooring | 0 | 118 (10.5%) | 13 (14.6%) | 105 (10.2%) | 0.21 | 1.51 | 0.62, 3.68 | 0.36 |  |
| - Carpeting or rug/mat flooring | 0 | 53 (4.7%) | 5 (5.6%) | 48 (4.7%) | 0.60 | 1.22 | 0.40, 3.69 | 0.72 |  |
| - Hardwood flooring | 0 | 275 (24.5%) | 17 (19.1%) | 258 (25%) | 0.25 | 0.71 | 0.37, 1.36 | 0.30 |  |
|  |  |  |  |  |  |  |  |  |  |
| **Bedroom** |  |  |  |  |  |  |  |  |  |
| Number of other subjects in the room | 0 | 1.0 [0.0, 1.0] | 1.0 [0.0, 1.0] | 1.0 [0.0, 1.0] | 0.66 | 1.11 | 0.74, 1.68 | 0.61 |  |
| Number of other children in the room | 0 | 0.0 [0.0, 0.0] | 0.0 [0.0, 0.0] | 0.0 [0.0, 0.0] | 0.06 | 1.81 | 1.00, 3.30 | 0.05 |  |
| Surface area (m²)* | 9 | 12 [11, 16] | 12 [10, 16] | 12 [11, 16] | 0.79 | 0.66 | 0.26, 1.64 | 0.37 |  |
| Surface area per subject (m²)* | 9 | 5 [3, 7] | 5 [3, 6.67] | 5 [3, 7] | 0.50 | 0.50 | 0.49, 1.40 | 0.47 |  |
|  |  |  |  |  |  |  |  |  |  |
| Bedding in natural feathers | 1 | 205 (18.3%) | 13 (14.6%) | 192 (18.6%) | 0.39 | 0.75 | 0.41, 1.37 | 0.35 |  |
| Double glazing in bedroom | 1 | 882 (78.8%) | 69 (77.5%) | 813 (78.9%) | 0.79 | 0.93 | 0.51, 1.69 | 0.80 |  |
| Presence of air humidifier | 1 | 61 (5.4%) | 6 (6.7%) | 55 (5.3%) | 0.62 | 1.28 | 0.49, 3.39 | 0.62 |  |
|  |  |  |  |  |  |  |  |  |  |
| Heating: |  |  |  |  |  |  |  |  |  |
| - Floor heating | 1 | 95 (8.5%) | 10 (11.2%) | 85 (8.2%) | 0.32 | 1.41 | 0.48, 4.10 | 0.53 |  |
| - Wall heating unit | 1 | 884 (78.9%) | 65 (73%) | 819 (79.4%) | 0.17 | 0.70 | 0.37, 1.32 | 0.27 |  |
| - Central heating | 1 | 592 (52.9%) | 47 (52.8%) | 545 (52.9%) | 1.00 | 1.00 | 0.60, 1.65 | 0.99 |  |
| - Electric heating | 1 | 321 (28.7%) | 20 (22.5%) | 301 (29.2%) | 0.22 | 0.70 | 0.40, 1.24 | 0.23 |  |
| - Fuel heating | 1 | 160 (14.3%) | 9 (10.1%) | 151 (14.6%) | 0.27 | 0.66 | 0.27, 1.61 | 0.36 |  |
| - Gas heating | 1 | 459 (41%) | 40 (44.9%) | 419 (40.6%) | 0.43 | 1.19 | 0.70, 2.04 | 0.52 |  |
|  |  |  |  |  |  |  |  |  |  |
| Flooring: |  |  |  |  |  |  |  |  |  |
| - Tiles flooring | 1 | 234 (20.9%) | 20 (22.5%) | 214 (20.8%) | 0.68 | 1.11 | 0.62, 1.98 | 0.73 |  |
| - Linoleum flooring | 1 | 182 (16.2%) | 19 (21.3%) | 163 (15.8%) | 0.18 | 1.45 | 0.72, 2.92 | 0.30 |  |
| - Carpeting or rug/mat flooring | 1 | 158 (14.1%) | 10 (11.2%) | 148 (14.4%) | 0.53 | 0.76 | 0.37, 1.56 | 0.45 |  |
| - Hardwood flooring | 1 | 551 (49.2%) | 39 (43.8%) | 512 (49.7%) | 0.32 | 0.79 | 0.48, 1.31 | 0.36 |  |

**Table S4. Nature and daily duration of meetings (minutes)**

|  | ***Missing values (N)*** | ***Distribution (all)*** | ***Distribution in infected subjects*** | | ***Distribution in non-infected subjects*** | | ***P (diff)*** | | ***OR*** | | ***(95% CI)*** | | ***P (OR)*** | |
| --- | --- | --- | --- | --- | --- | --- | --- | --- | --- | --- | --- | --- | --- | --- |
| **Duration of meetings:** |  |  | |  | |  | |  | |  |  |  | |  |
| - All* | 0 | 7.08 [6.49, 7.64] | | 6.98 [6.52, 7.54] | | 7.08 [6.49, 7.66] | | 0.42 | | 0.88 | 0.69, 1.13 | 0.33 | |  |
| - At home* | 0 | 6.2 [5.63, 6.63] | | 6.17 [5.61, 6.64] | | 6.2 [5.64, 6.62] | | 0.43 | | 0.87 | 0.79, 0.97 | < 0.02 | |  |
| - At school* | 0 | -6.91 [-6.91, 5.13] | | -6.91 [-6.91, 5.97] | | -6.91 [-6.91, 5.08] | | 0.78 | | 1.00 | 0.96, 1.04 | 0.83 | |  |
| - At work* | 0 | 2.25 [-6.91, 5.93] | | 0.11 [-6.91, 5.34] | | 2.3 [-6.91, 5.96] | | 0.34 | | 0.99 | 0.96, 1.03 | 0.64 | |  |
| - In transports* | 0 | -6.91 [-6.91, -6.91] | | -6.91 [-6.91, -6.91] | | -6.91 [-6.91, -6.91] | | 0.53 | | 0.98 | 0.93, 1.04 | 0.50 | |  |
|  |  |  | |  | |  | |  | |  |  |  | |  |
| With subjects aged: |  |  | |  | |  | |  | |  |  |  | |  |
| - 0-10 years* | 0 | 3.62 [-6.91, 5.48] | | 3.98 [-6.91, 6.06] | | 3.51 [-6.91, 5.36] | | 0.13 | | 1.02 | 0.97, 1.07 | 0.45 | |  |
| - 10-30 years* | 0 | 5.25 [3.62, 6.18] | | 4.97 [2.41, 5.92] | | 5.27 [3.75, 6.19] | | < 0.02 | | 0.94 | 0.89, 0.99 | < 0.02 | |  |
| - 30-50 years* | 0 | 5.9 [4.95, 6.49] | | 5.79 [4.84, 6.43] | | 5.91 [4.97, 6.49] | | 0.37 | | 0.95 | 0.86, 1.06 | 0.37 | |  |
| - 50-70 years* | 0 | 4.80 [3.53, 5.58] | | 4.66 [2.81, 5.47] | | 4.81 [3.58, 5.58] | | 0.08 | | 0.94 | 0.89, 1.00 | < 0.04 | |  |
| - > 70 years* | 0 | -6.91 [-6.91, 3.77] | | -6.91 [-6.91, 3.62] | | -6.91 [-6.91, 3.77] | | 0.33 | | 0.98 | 0.93, 1.02 | 0.33 | |  |

**Table S5. Geographic area**

|  | ***Missing values (N)*** | ***Distribution (all)*** | ***Distribution in infected subjects*** | | ***Distribution in non-infected subjects*** | | ***P (diff)*** | ***OR*** | | ***(95% CI)*** | | ***P (OR)*** | |
| --- | --- | --- | --- | --- | --- | --- | --- | --- | --- | --- | --- | --- | --- |
| **Activity near habitation** |  |  | |  | |  |  | |  |  |  | |  |
| Agricultural land | 0 | 572 (51.0%) | | 54 (60.7%) | | 518 (50.2%) | 0.06 | | 1.53 | 0.9, 2.61 | 0.12 | |  |
| Presence of farming: |  |  | |  | |  |  | |  |  |  | |  |
| - Any | 0 | 61 (5.4%) | | 5 (5.6%) | | 56 (5.4%) | 0.81 | | 1.04 | 0.36, 2.96 | 0.95 | |  |
| - Poultry | 0 | 50 (4.5%) | | 2 (2.2%) | | 48 (4.7%) | 0.42 | | 0.47 | 0.12, 1.88 | 0.29 | |  |
| - Pork | 0 | 14 (1.2%) | | 3 (3.4%) | | 11 (1.1%) | 0.09 | | 3.24 | 0.9, 11.68 | 0.07 | |  |
| Factory | 0 | 249 (22.2%) | | 26 (29.2%) | | 223 (21.6%) | 0.11 | | 1.50 | 0.81, 2.78 | 0.20 | |  |
|  |  |  | |  | |  |  | |  |  |  | |  |
| **Demography** |  |  | |  | |  |  | |  |  |  | |  |
| Proportion of inhabitants: |  |  | |  | |  |  | |  |  |  | |  |
| - Living at the same place 5 years ago | 0 | 0.67 [0.60, 0.73] | | 0.68 [0.61, 0.73] | | 0.67 [0.60, 0.72] | 0.44 | | 3.35 | 0.24, 46.61 | 0.37 | |  |
| - Executive, intellectual profession | 0 | 0.08 [0.05, 0.13] | | 0.08 [0.04, 0.14] | | 0.08 [0.05, 0.13] | 0.40 | | 0.51 | 0.02, 15.09 | 0.70 | |  |
| - Middle class | 0 | 0.16 [0.14, 0.19] | | 0.16 [0.13, 0.19] | | 0.16 [0.14, 0.19] | 0.33 | | 0.02 | 0.00, 22.37 | 0.29 | |  |
| - Employee | 0 | 0.18 [0.15, 0.21] | | 0.18 [0.14, 0.21] | | 0.18 [0.15, 0.21] | 0.52 | | 0.09 | 0.00, 47.91 | 0.45 | |  |
| - Working class | 0 | 0.14 [0.10, 0.19] | | 0.15 [0.10, 0.19] | | 0.14 [0.10, 0.19] | 0.98 | | 1.43 | 0.02, 97.65 | 0.87 | |  |
| - Working in same commune | 0 | 0.19 [0.13, 0.39] | | 0.16 [0.11, 0.35] | | 0.20 [0.13, 0.40] | 0.18 | | 0.31 | 0.06, 1.67 | 0.17 | |  |
| - Working in same department | 0 | 0.26 [0.14, 0.45] | | 0.27 [0.18, 0.46] | | 0.26 [0.14, 0.45] | 0.33 | | 1.66 | 0.38, 7.28 | 0.50 | |  |
| - Using public transport to go to work | 0 | 0.05 [0.02, 0.11] | | 0.06 [0.02, 0.12] | | 0.05 [0.02, 0.11] | 0.90 | | 1.13 | 0.13, 10.00 | 0.91 | |  |
|  |  |  | |  | |  |  | |  |  |  | |  |
| Proportion of households with: |  |  | |  | |  |  | |  |  |  | |  |
| - Persons living alone | 0 | 0.28 [0.22, 0.41] | | 0.26 [0.21, 0.43] | | 0.28 [0.22, 0.41] | 0.44 | | 0.65 | 0.10, 4.31 | 0.65 | |  |
| - Families with children | 0 | 0.30 [0.21, 0.37] | | 0.32 [0.20, 0.38] | | 0.30 [0.22, 0.37] | 0.37 | | 2.44 | 0.17, 35.76 | 0.52 | |  |
| - Couples with no children | 0 | 0.28 [0.22, 0.33] | | 0.28 [0.22, 0.33] | | 0.28 [0.22, 0.33] | 0.70 | | 0.26 | 0.00, 13.75 | 0.50 | |  |
|  |  |  | |  | |  |  | |  |  |  | |  |
| Proportion of inhabitants > 15 years: |  |  | |  | |  |  | |  |  |  | |  |
| - With no diploma | 0 | 0.55 [0.46, 0.64] | | 0.56 [0.43, 0.66] | | 0.55 [0.46, 0.63] | 0.57 | | 1.65 | 0.2, 13.8 | 0.64 | |  |
| - Not attending higher education | 0 | 0.08 [0.05, 0.14] | | 0.08 [0.05, 0.17] | | 0.08 [0.05, 0.14] | 0.75 | | 0.59 | 0.03, 10.06 | 0.71 | |  |
|  |  |  | |  | |  |  | |  |  |  | |  |

| Proportion of habitations: |  |  |  |  |  |  |  |  |
| --- | --- | --- | --- | --- | --- | --- | --- | --- |
| - Houses (vs. other habitations) | 0 | 0.67 [0.17, 0.90] | 0.63 [0.13, 0.91] | 0.67 [0.19, 0.90] | 0.70 | 0.79 | 0.37, 1.67 | 0.53 |
| - With surface > 40 m2 | 0 | 0.06 [0.03, 0.12] | 0.06 [0.03, 0.11] | 0.05 [0.03, 0.13] | 1.00 | 0.56 | 0.08, 3.86 | 0.56 |
| - With surface > 100 m2 | 0 | 0.26 [0.14, 0.45] | 0.30 [0.13, 0.43] | 0.26 [0.14, 0.45] | 0.86 | 0.97 | 0.22, 4.18 | 0.96 |
| - Owned by inhabitants | 0 | 0.64 [0.44, 0.78] | 0.63 [0.39, 0.80] | 0.64 [0.44, 0.78] | 0.77 | 0.72 | 0.21, 2.56 | 0.62 |
| - Social housing | 0 | 0.07 [0.01, 0.17] | 0.07 [0.01, 0.17] | 0.07 [0.01, 0.17] | 0.86 | 1.75 | 0.38, 8.07 | 0.47 |
| - With collective heating system | 0 | 0.06 [0.02, 0.25] | 0.05 [0.02, 0.38] | 0.06 [0.02, 0.25] | 0.65 | 1.57 | 0.51, 4.82 | 0.43 |
| - With private electric heating | 0 | 0.24 [0.13, 0.34] | 0.21 [0.12, 0.32] | 0.24 [0.13, 0.34] | 0.26 | 0.33 | 0.06, 1.77 | 0.20 |
|  |  |  |  |  |  |  |  |  |
| Proportion of car ownership | 0 | 0.86 [0.74, 0.92] | 0.88 [0.77, 0.94] | 0.86 [0.74, 0.92] | 0.25 | 1.65 | 0.27, 9.97 | 0.59 |
| Annual income (k€) | 0 | 22.1 [19.5, 26.6] | 22.1 [19.7, 27.5] | 22.1 [19.5, 26.6] | 0.48 | 1.00 | 0,97 1.02 | 0.67 |
