# Supplementary material for: Risk factors of pandemic influenza A/H1N1 in a prospective household cohort in the general population: results from the CoPanFlu-France cohort
Source: Influenza Other Respir Viruses. 2014 Nov 10;9(1):43–50. doi: 10.1111/irv.12294 (PMC4280818; doi:10.1111/irv.12294)
Supplement: Supplementary file 2 — Data S2. Multivariable selection procedure and results (Tables S6–S18). [file irv0009-0043-sd2.docx]

**Risk factors of pandemic influenza A/H1N1 in a prospective household cohort in the general population: results from the CoPanFlu-France cohort**

**Multivariable selection procedure**

As mentioned in a previous article, an impressive amount of data was collected in this cohort and the simultaneous analysis of many covariates with logistic regression modeling is likely to favor artifactual associations (1). Moreover, most variable selection strategies aim to identify the *best* multivariable model according to an information criterion, and therefore discard the insightful information from covariates fitting other relevant models, insinuating a sole correct model could be meaningful. As the number of candidate multivariable models grows exponentially with the number of possible covariates, the likelihood of selecting a suboptimal model and missing information brought by other covariates must be considered (2). To limit the risk of adventitious results, we therefore developed a 2-step analysis consisting in (i) selecting a limited set of relevant covariates with the use of multimodel selection and (ii) identifying factors independently associated with infections among these relevant covariates. Our analysis plan is summarized hereafter:

- Control of intra-household correlation

Risk factors for infection were studied via logistic regression with the use of generalized estimating equations (GEE) accounting for the correlation between responses of subjects living in the same household. The quasi-likelihood under Independence Model Criterion (QIC) was used as information criterion to select models (3).

- Absence of univariable screening

The selection procedure was run among all covariates, regardless of univariable regression results, thus avoiding to reject potentially meaningful factors whose association with H1N1v infection would be linked to an improperly controlled confounder (4).

- Multimodel selection

A first set of relevant covariates was selected using multimodel selection: these covariates were not necessarily present in the multivariable model with the best QIC, but in any of the most insightful multivariable models, according to this information criterion.

Robustness of this multimodel selection was enhanced by multiple imputation with two-stage bootstrap resampling, with respect to the household-clustered nature of data (5). QIC for all selected models was averaged over 30 imputed datasets with 20 bootstrap resamplings each.

Candidate samples of 10 covariates were iteratively drawn from the whole set of covariates with the use of a genetic algorithm ensuring that covariates already selected in multivariable models with the best information criterion had an increased probability to be drawn again. In each sample of candidate covariates, a stepwise backward/forward QIC-based model selection was carried out.

All selected models were sorted according to their QIC and were assigned a relative weight according to their relevancy, as defined by Burnham and Anderson for the AIC (6,7). This weight, whose sum over all models is 1, can be interpreted as the probability that a model is the best model for the data among all those evaluated, according to the Kullback-Leibler information theory (7). Models were considered as meaningful if their QIC weight was over 0.001 and covariates entering any of these selected models were defined as relevant.

- Control of the pre-epidemic titer

The decision of whether or not to include the pre-epidemic titer as a covariate in risk factors analyses is controversial: although several studies do so (8,9), a 4-fold rise of titer after infection may be less likely to occur in subjects with an already elevated titer (10), thus yielding a regression toward the mean bias when seroconversions are used to define infections. To handle this issue, we conducted a sensitivity analysis and carried out two multimodel selections as previously described, with and without this covariate respectively.

- Final model selection

A stepwise backward/forward multivariable model selection procedure was finally conducted to identify factors independently associated with infections. P < 0.05 was required for all covariates entering the final model. This model selection was run among all covariates defined as relevant by either multimodel selection. As including the pre-epidemic titer in this final model selection had no impact on the list of other selected covariates, we decided to keep this covariate in the final model.

**Multimodel selection results: list of models with QIC weight > 0.001**

1. Multimodel selection 1 (excluding pre-epidemic titer as a covariate)

- **Table S6: multimodel selection 1, selected model 1**

|  | **OR** | **95% CI** | **P** |
| --- | --- | --- | --- |
| Any cardiovascular disease | 1.58 | 0.95, 2.63 | 0.08 |
| Household members covering mouth while coughing or sneezing (per 10% increase) | 0.96 | 0.93, 0.99 | < 0.03 |
| Influenza vaccination (2010) | 0.63 | 0.25, 1.56 | 0.31 |
| *alpha (intra-household correlation parameter)* | *0.44* |  |  |

QIC weight: 0.454

- **Table S7: multimodel selection 1, selected model 2**

|  | **OR** | **95% CI** | **P** |
| --- | --- | --- | --- |
| Green tea consumption ≥ 2/week | 0.50 | 0.13, 1.95 | 0.32 |
| Household daily frequency of handwashing | 0.95 | 0.88, 1.02 | 0.17 |
| *alpha (intra-household correlation parameter)* | *0.44* |  |  |

QIC weight: 0.235

- **Table S8: multimodel selection 1, selected model 3**

|  | **OR** | **95% CI** | **P** |
| --- | --- | --- | --- |
| History of ILI during 2009 pandemic wave | 0.59 | 0.17, 2.04 | 0.40 |
| Pandemic vaccination (2009) | 0.56 | 0.23, 1.37 | 0.21 |
| *alpha (intra-household correlation parameter)* | *0.50* |  |  |

QIC weight: 0.156

- **Table S9: multimodel selection 1, selected model 4**

|  | **OR** | **95% CI** | **P** |
| --- | --- | --- | --- |
| Daily frequency of handwashing | 0.98 | 0.93, 1.05 | 0.62 |
| Pandemic vaccination (2009) | 0.34 | 0.07, 1.65 | 0.18 |
| *alpha (intra-household correlation parameter)* | *0.47* |  |  |

QIC weight: 0.097

- **Table S10: multimodel selection 1, selected model 5**

|  | **OR** | **95% CI** | **P** |
| --- | --- | --- | --- |
| Number of other children sharing the bedroom | 1.2 | 0.49, 2.92 | 0.69 |
| Influenza vaccination (2010) coverage in household (per 10% increase) | 0.99 | 0.94, 1.06 | 0.88 |
| *alpha (intra-household correlation parameter)* | *0.46* |  |  |

QIC weight: 0.031

- **Table S11: multimodel selection 1, selected model 6**

|  | **OR** | **95% CI** | **P** |
| --- | --- | --- | --- |
| Any cardiovascular disease | 1.53 | 0.74, 3.15 | 0.25 |
| Influenza vaccination (2010) | 0.60 | 0.20, 1.80 | 0.37 |
| *alpha (intra-household correlation parameter)* | *0.46* |  |  |

QIC weight: 0.018

- **Table S12: multimodel selection 1, selected model 7**

|  | **OR** | **95% CI** | **P** |
| --- | --- | --- | --- |
| History of asthma | 1.44 | 0.45, 4.60 | 0.54 |
| Green tea consumption ≥ 2/week | 0.51 | 0.18, 1.41 | 0.20 |
| *alpha (intra-household correlation parameter)* | *0.47* |  |  |

QIC weight: 0.005

- **Table S13: multimodel selection 1, selected model 8**

|  | **OR** | **95% CI** | **P** |
| --- | --- | --- | --- |
| History of asthma | 1.72 | 0.67, 4.41 | 0.26 |
| Influenza vaccination (2010) | 0.70 | 0.25, 1.93 | 0.49 |
| *alpha (intra-household correlation parameter)* | *0.50* |  |  |

QIC weight: 0.003

- **Table S14: multimodel selection 1, selected model 9**

|  | **OR** | **95% CI** | **P** |
| --- | --- | --- | --- |
| Green tea consumption ≥ 2/week | 0.40 | 0.15, 1.06 | 0.07 |
| *alpha (intra-household correlation parameter)* | *0.48* |  |  |

QIC weight: 0.001

1. Multimodel selection 2 (with pre-epidemic titer as a covariate)

- **Table S15: multimodel selection 2, selected model 1**

|  | **OR** | **95% CI** | **P** |
| --- | --- | --- | --- |
| Pre-epidemic titer × 2 | 0.49 | 0.32, 0.76 | < 0.01 |
| Influenza vaccination (2010) | 0.81 | 0.14, 4.51 | 0.81 |
| *alpha (intra-household correlation parameter)* | *0.43* |  |  |

QIC weight: 0.43

- **Table S16: multimodel selection 2, selected model 2**

|  | **OR** | **95% CI** | **P** |
| --- | --- | --- | --- |
| History of asthma | 2.53 | 1.18, 5.40 | < 0.02 |
| Number of other children sharing the bedroom | 1.57 | 0.59, 4.18 | 0.36 |
| Pre-epidemic titer × 2 | 0.49 | 0.34, 0.70 | < 0.001 |
| *alpha (intra-household correlation parameter)* | *0.43* |  |  |

QIC weight: 0.245

- **Table S17: multimodel selection 2, selected model 3**

|  | **OR** | **95% CI** | **P** |
| --- | --- | --- | --- |
| History of asthma | 2.04 | 1.02, 4.09 | < 0.05 |
| Household daily frequency of handwashing | 0.94 | 0.85, 1.02 | 0.15 |
| Pre-epidemic titer × 2 | 0.47 | 0.31, 0.81 | < 0.01 |
| *alpha (intra-household correlation parameter)* | *1.61* |  |  |

QIC weight: 0.217

- **Table S18: multimodel selection 2, selected model 4**

|  | **OR** | **95% CI** | **P** |
| --- | --- | --- | --- |
| History of asthma | 2.38 | 0.94, 6.02 | 0.07 |
| Household members covering mouth while coughing or sneezing (per 10% increase) | 0.96 | 0.93, 0.99 | < 0.03 |
| Pre-epidemic titer × 2 | 0.51 | 0.35, 0.74 | < 0.001 |
| *alpha (intra-household correlation parameter)* | *0.44* |  |  |

QIC weight: 0.108

**References**

1. Hsueh H, Chen JJ, Kodell RL. Comparison of methods for estimating the number of true null hypotheses in multiplicity testing. J Biopharm Stat. 2003 Nov;13(4):675–89.

2. Madigan D, Raftery AE. Model Selection and Accounting for Model Uncertainty in Graphical Models Using Occam’s Window. J Am Stat Assoc. 1994 Dec;89(428):1535–46.

3. Pan W. Akaike’s Information Criterion in Generalized Estimating Equations. Biometrics. 2001 Mar 1;57(1):120–5.

4. Sun G, Shook T, Kay G. Inappropriate use of bivariable analysis to screen risk factors for use in multivariable analysis. J Clin Epidemiol. 1996 Aug;49(8):907–16.

5. Field CA, Welsh AH. Bootstrapping clustered data. J R Stat Soc Ser B Stat Methodol. 2007 Jun 1;69(3):369–90.

6. Burnham KP, Anderson DR. Multimodel Inference Understanding AIC and BIC in Model Selection. Sociol Methods Res. 2004 Nov 1;33(2):261–304.

7. Burnham KP, Anderson DR. Model Selection and Multi-Model Inference: A Practical Information-Theoretic Approach. Springer; 2002. 512 p.

8. Chao D-Y, Cheng K-F, Li T-C, Wu T-N, Chen C-Y, Tsai C-A, et al. Factors associated with infection by 2009 pandemic H1N1 influenza virus during different phases of the epidemic. Int J Infect Dis IJID Off Publ Int Soc Infect Dis. 2011 Oct;15(10):e695–e701.

9. Chen MIC, Lee VJM, Lim W-Y, Barr IG, Lin RTP, Koh GCH, et al. 2009 influenza A(H1N1) seroconversion rates and risk factors among distinct adult cohorts in Singapore. JAMA J Am Med Assoc. 2010 Apr 14;303(14):1383–91.

10. Petrie JG, Ohmit SE, Johnson E, Cross RT, Monto AS. Efficacy Studies of Influenza Vaccines: Effect of End Points Used and Characteristics of Vaccine Failures. J Infect Dis. 2011 Mar 4;
